# Water Dynamics of Superacid Aromatic Proton Exchange Membranes for Fuel Cell Applications

**DOI:** 10.1021/acs.macromol.4c02925

**Published:** 2025-02-20

**Authors:** Zitan Huang, Sol Mi Oh, Karen I. Winey, Michael A. Hickner

**Affiliations:** † Department of Materials Science and Engineering, The Pennsylvania State University, University Park, Pennsylvania 16802, United States; ‡ Department of Materials Science and Engineering, University of Pennsylvania, Philadelphia, Pennsylvania 19104, United States; § Department of Chemical and Biomolecular Engineering, University of Pennsylvania, Philadelphia, Pennsylvania 19104, United States; ∥ Department of Chemical Engineering and Materials Science, Michigan State University, East Lansing, Michigan 48824, United States

## Abstract

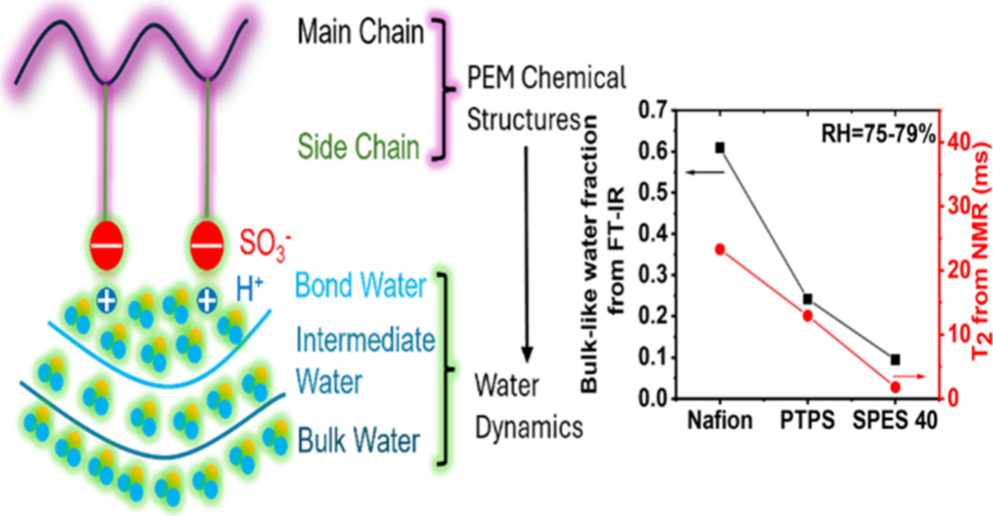

Proton exchange membranes (PEMs) with high conductivity are of critical importance for the development of fuel cells, electrolyzers, and other electrochemical technologies. In this research, poly(1,1,2,2-tetrafluoro-2-phenoxyethane-1-sulfonic acid) (PTPS) with an aromatic polymer main chain and a perfluorinated superacidic polymer side chain was synthesized. The water dynamics of PTPS were characterized across various length scales using a combination of Fourier-transform infrared spectroscopy (FTIR) and nuclear magnetic resonance (NMR) and compared with Nafion, a standard perfluorinated PEM, and sulfonated poly(ether sulfone) (SPES 40), an aromatic PEM without perfluorinated superacid side chains. The *T*_1_ and *T*_2_ relaxation times of water in the samples probed by NMR increase from SPES 40 to PTPS to Nafion, indicating that the local motion of the water molecules becomes faster. This trend corresponds well with the relative fraction of bulk-like water determined using FTIR. At larger length scales, the diffusion coefficient of water was characterized using pulsed-field gradient NMR (PFG-NMR). At a longer diffusion time (Δ = 100 ms), PTPS has a smaller diffusion coefficient compared with both Nafion and SPES 40, due to restricted diffusion, and this effect is also evident in the proton conductivity of the hydrated membranes. From this comparison, it is apparent that the aromatic backbone and side chain type greatly influence the water dynamics in PEMs at various length scales and the water dynamics significantly impact the bulk proton conductivity. These insights will lead to new designs for aromatic PEMs and help to identify bottlenecks in current materials.

## Introduction

Fuel cells convert the chemical energy of a fuel (such as hydrogen or methanol) into electrical energy through an electrochemical process that can occur at low temperatures across a range of conditions.^1^ Due to the clean nature of this technology and its other advantages such as low emissions, quiet operation, and few moving parts, fuel cells have wide-ranging applications in portable electronic devices,^2^ stationary power,^3^ and transportation.^4^ Among the various types of fuel cells, proton exchange membrane fuel cells (PEMFCs) are one of the most promising variants for widespread adoption.^5^ In these systems, the proton exchange membrane (PEM) is a semipermeable membrane that concurrently conducts protons between the electrodes and prevents the permeation of unreacted hydrogen and oxygen gases between the anode and cathode.^6^ To meet the performance requirements of fuel cell applications in general, PEMs are required to have high conductivity, reasonably good mechanical properties, and sufficient thermal and chemical stability.^7^

Perfluorosulfonic acid (PFSA) polymers (e.g., Nafion, Aquivion, and others) are the standard membranes in many electrochemical technologies because of their high conductivity,^8^ superior chemical stability, and good mechanical properties, originating from their unique perfluorinated chemical structure and phase-separated morphology when hydrated.^9−11^ However, due to the harsh conditions for PFSA synthesis and processing,^12^ high degree of gas permeation, ease of dehydration at elevated temperatures, and concerns about perfluorinated alkyl substances (PFAS), many researchers have turned to aromatic polymers containing little or no organofluoride as the backbone materials for PEMs.

Due to the rigidity of the main chain of aromatic high *T*_g_ polymers, these materials show high thermal and chemical stability and have a wide range of chemical structures.^7^ Most hydrocarbon-based PEMs show significantly lower proton conductivity compared with PFSAs because of the low acidity of the aromatic sulfonic acid and a lower degree of phase segregation in these systems.^13^ Therefore, many researchers have turned to block copolymers,^14−16^ graft copolymers,^17^ cross-linking polymer systems,^18,19^ and densely sulfonated polymers,^20,21^ to improve the conductivity of non-PFSA PEMs. Attaching a perfluoro alkyl superacidic side chain to a hydrocarbon aromatic polymer backbone can be beneficial for improving proton conductivity without requiring a fully perfluorinated polymer.^22−24^

The water dynamics and hydration environment at various length scales greatly influence the proton conductivity and transport mechanisms in the PEMs. Thus, understanding water behavior on different length scales is of critical importance. Both NMR relaxometry and diffusion were used to characterize the water environments in PEMs.

NMR relaxation, as quantified by the *T*_1_ and *T*_2_ of water or D_2_O, is a commonly used method to quantify water dynamics in a range of environments. *T*_1_ represents the spin–lattice relaxation time, while *T*_2_ represents the spin–spin relaxation time, and both of them are mathematically connected to the motions of water molecules^25^ (such as rotational diffusion^26^), due to the similarity in the time scale of the molecular motion and the NMR measurement.^27^ Therefore, many research groups have turned to NMR relaxation measurement to probe the water dynamics in PEMs, especially in Nafion.^27,28^ Zawodzinski et al.^29^ showed that the water dynamics in Nafion were restricted due to the interaction of the water with the polymer membrane as evidenced by a much smaller *T*_1_ value compared with that of bulk water. Similarly, by systematically analyzing the ^1^H NMR *T*_1_ and diffusion coefficient data at various temperatures and water uptake levels in Nafion samples, Hammer et al. established a comprehensive picture of the water environment in Nafion which is composed of two hydration shells and bulk water.^30^ Their finding is in good agreement with Fourier-transform infrared (FTIR) spectroscopy measurements, as discussed below. Lee et al. compared the ^2^H NMR *T*_1_ and *T*_2_ for Aquivion, Nafion, and sulfonated Radel (aromatic polymer) and concluded that the water dynamics was much slower in Radel compared with Aquivion and Nafion due to the stronger interaction between the ionic groups and the water molecules.^31^

At length scales greater than the hydration interactions, the water dynamics in PEMs can be characterized by measuring the water self-diffusion coefficient using pulsed-field gradient (PFG) NMR. As this measurement obtains diffusion coefficient by measuring the diffusional dephasing under a gradient-encoded magnetization, PFG-NMR reports the diffusion coefficient at varying length scales (from nanometer to millimeter scale) by simply changing the diffusion time in the pulse-field sequence.^32,33^ Together with other morphological measurements, the diffusion coefficient measurement at different length scales can provide a comprehensive picture of water motion in PEMs.^32,34^ Additionally, by combining the water diffusion coefficient from PFG-NMR and diffusion coefficient converted from proton conductivity, Kreuer et al. provide indirect evidence of the Grotthus mechanism for proton conduction in Nafion.^35^

The OD stretch peak of dilute HOD in H_2_O as probed by Fourier-transform infrared (FTIR) spectroscopy can be used as a probe to study the hydrogen-bonding network of water, which is influenced by neighboring charged species and molecular structuring.^36−38^ This FTIR signature has a high sensitivity of its peak center frequency to the water environment and is a well-defined singular stretching mode without the overlap of other vibrational modes from H_2_O and D_2_O. Moilanen et al.^37^ studied water environments in Nafion and found that there are two populations of water: headgroup-associated water and bulk-like water. The relative fraction of each population was obtained after peak deconvolution, which confirmed that the bulk-like water fraction increased with the hydration number. Our previous work also performed systematic investigations on hydrocarbon-based PEM materials with different side chain structures to examine the effect of backbone polarity and acidity of the sulfonic acid group on the water environment.^39−41^ We showed previously that this FTIR technique has broad applicability across different classes of membranes.

**Figure 1:**
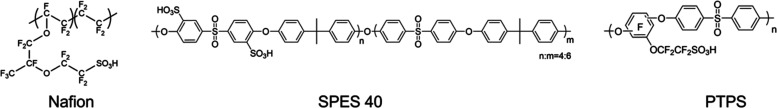
Chemical structures of Nafion, sulfonated poly(ether sulfone) (SPES 40), and poly(1,1,2,2-tetrafluoro-2-phenoxyethane-1-sulfonic acid) (PTPS).

In this research, an aromatic backbone polymer with a superacid side chain was synthesized. In this context, we consider superacid acidic groups that have an acidity higher than sulfuric acid. Fluorinated acids, such as those on PFSAs, are one such example of superacids. Together with two other commercially available PEMs (chemical structures shown in Figure 1), their water dynamics were characterized at various length scales using NMR relaxation measurements, PFG-NMR diffusion measurements, and FTIR. Through a combination of these characterization methods, the effect of the chemical structures of PEMs on the water dynamics is systematically investigated.

## Experimental Section

### Materials

4,4′-Sulfonyl diphenol (98%), K_2_CO_3_ (≥99%), dimethylacetamide (DMAc, anhydrous, 99.8%), 2,3,5,6-tetrafluoro phenol (95%), dimethyl sulfoxide (DMSO, anhydrous, ≥99.9%), sodium hydrosulfite (≥82%), acetonitrile (anhydrous, 99.8%), toluene (anhydrous, 99.8%), cesium carbonate (99%), sodium bicarbonate (≥99.5%), hydrogen peroxide solution (30 wt % in H_2_O), isopropyl alcohol (98%), and hydrochloric acid (37%) were purchased from Millipore Sigma, Inc. (St. Louis, MO). 1,2-Dibromotetrafluoroethane was purchased from SynQuest Lab, Inc. (Alachua, FL). Nafion NR211 was purchased from Ion Power, Inc. (Tyrone, PA). SPES 40 was purchased from Yanjin Technology Inc. (Tianjin, China).

### PTPS Synthesis

**Scheme 1:**
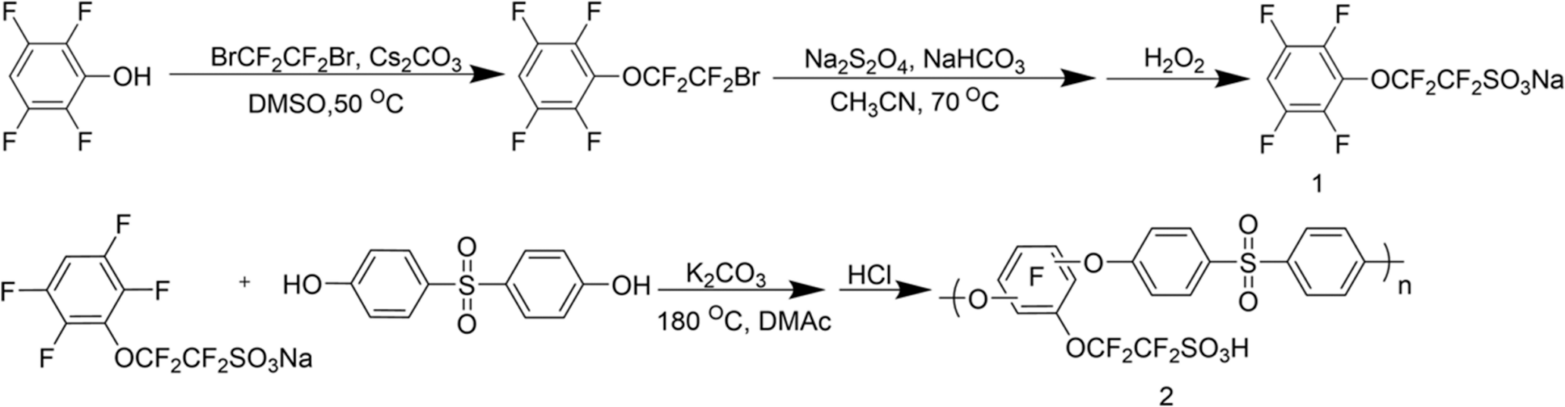
Synthesis of PTPS

The synthesis of PTPS is shown in Scheme 1. The synthesis procedure of the monomer, 1,2,2-tetrafluoro-2-(2,3,5,6-tetrafluorophenoxy)ethane-1-sulfonate (TFTS, structure 1 in Scheme 1) was based on a previous report.^42^ During the polymer synthesis process, 4,4′-sulfonyl diphenol (1 equiv), anhydrous DMAc, anhydrous toluene, and K_2_CO_3_ (1.5 equiv) were added into a round-bottom flask equipped with a Dean–Stark trap and a reflux condenser under an argon environment. The mixture was vigorously stirred at 170 °C for 4 h until no more water was formed in the Dean–Stark trap. The mixture was then cooled to 150 °C, and the solution of TFTS (1 equiv) in DMAc was added into the system through a syringe and the system was further stirred for 18 h until the reaction mixture became viscous. The obtained viscous mixture was cooled to room temperature and then precipitated in isopropyl alcohol. The precipitate was then washed with water four times. A brown solid was obtained after drying at 80 °C under vacuum. To form a membrane, the dried samples were dissolved in DMSO (0.1 g/mol), cast into a flat Teflon mold, and dried at 70 °C for 72 h to obtain a dark brown membrane approximately 33 μm in thickness. The obtained membrane was immersed in 3 M HCl solution for 48 h at 40 °C to convert the polymer to its proton form and then the sample was further washed with water and dried under vacuum. The chemical structure of polymers and monomers was measured by ^1^H NMR and ^19^F NMR using dimethyl sulfoxide-d6 (DMSO-*d*_6_) as a solvent.

### Water Uptake

The hydrated masses of Nafion, SPES 40, and PTPS were measured after equilibration at a given relative humidity (RH) (95, 75, and 50%) for 3 weeks. Their dry mass was then measured after drying at 80 °C under vacuum for 24 h. The water uptake was calculated by using the following equation:
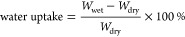
where, *W*_wet_ represents the wet mass of the membrane after equilibration, and *W*_dry_ represents the dry mass of the membrane. The error range for water uptake is approximately ±3% based on an assessment of the propagation of uncertainty of the measurement.

### Ion Exchange Capacity (IEC)

The IEC of PTPS was obtained via ^1^H NMR (Figure S1b) through the following equation:
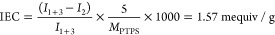
where *I*_1+3_ represents the relative peak intensity of the 1 + 3 peak of the ^1^H NMR spectrum, *I*_2_ represents the peak intensity of the 2 peak of the ^1^H NMR spectrum, and *M*_PTPS_ represents the molecular weight of the repeat unit of the PTPS polymer.

The IEC of PTPS was determined using titration; 0.1 M NaOH solution was used as a titrant. During the titration process, the pH was monitored with a SevenExcellence pH meter S400 (Mettler-Toledo, LLC, Columbus, OH). The IEC was calculated using the following equation:
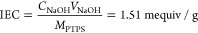


The IEC of PTPS obtained from titration is similar to that obtained by ^1^H NMR, which shows the viability of both methods to obtain accurate IEC values. Moreover, this titration and ^1^H NMR comparison demonstrate that there were no or very few ionic groups that were inaccessible to hydration.

### Fourier-Transform Infrared Spectroscopy

The membranes were hydrated under targeted relative humidity (RH) conditions using salt solutions. A wide range of RH conditions can be achieved, depending on the concentration and type of salt. For instance, 8.4, 20, 23.5, 28, and 35.3 wt % LiCl aqueous solutions form relative humidity (RH) conditions of 97, 67, 60, 60, 51, and 30% at room temperature, respectively.^43,44^ The saturated NaCl and KCl aqueous solutions were also used to create 79 and 87% RH at room temperature, respectively. We note that the solvent used for the salt solutions was 15% HOD in H_2_O, which is used to study the polymer–water interactions by observing the OD stretch peak of HOD molecules. Each salt solution was placed at the bottom of a 15 mL conical centrifuge tube, a porous sponge was inserted over the solution in the middle of the tube, and a piece of membrane with a plastic coverslip support was vertically placed on the sponge to separate it from the solution. The tube was then capped and sealed with parafilm to allow the membrane to be hydrated under specific RH conditions for more than 2 weeks. The mass of the membrane was measured during the hydration process by quickly removing the sample from the tube to confirm that the membranes reached equilibrium when the hydrated mass of the membrane stabilized. A Nicolet iS20 Midinfrared FTIR spectrometer (ThermoFisher) with a DTGS detector and interferometer with KBr/Ge-coated beamsplitter and dynamic alignment was used to measure FTIR spectra of the hydrated membranes. The spectral resolution and scan number were 2 cm^–1^ and 100, respectively. The spectra were measured in the attenuated total reflection (ATR) mode with an AR-coated diamond crystal (iD7 ATR accessory, ThermoFisher). Membranes placed on top of the ATR crystal were covered to maintain the desired hydrated state during the measurement. To test for repeatability, the FTIR data were measured at least 3 times consecutively (50 scans each) and there was no discernible change in the spectra, indicating that the hydrated state was maintained during the FTIR experiment. The ATR correction was carried out for all FTIR data after measurement. FTIR spectra from the corresponding membrane hydrated with H_2_O were taken as the background. After background subtraction, baseline correction was performed by setting the intensity of both ends of the OD stretch peak to 0. The OD stretch peak appeared at slightly different wavenumbers for each sample, 2300–2750 cm^–1^ for Nafion, 2350–2750 cm^–1^ for PTPS, and 2400–2700 cm^–1^ for SPES 40.

### Electrochemical Impedance Spectroscopy (EIS)

The proton conductivity, σ, of the PEMs was measured using a Solartron Analytical (Farnborough, United Kingdom) Modulab XM MTS spectrometer. The humidity and temperature were controlled by an SM-1.0 benchtop environmental chamber (Thermotron Industries, Holland, Michigan). A rectangular membrane sample with well-defined dimensions of surface area (a few mm^2^) and thickness (a few tens of μm) was placed on the glass slide to connect both ends to the impedance analyzer. Al foil and binder clips were used to make a good contact between the sample and analyzer. After achieving a target RH condition and temperature in the humidity chamber after sample loading, the complex dielectric constant, ε*, as a function of frequency (10^–2^–10^6^ Hz) was repeatedly measured until the data no longer changed, indicating steady-state. After obtaining the data, the sample dimensions were quickly remeasured (<20 s) and used to calculate the conductivity. Finally, the complex conductivity, σ*, was extracted using the measured ε* and the real part of σ*, namely σ*′* (=ωε″), at the frequency where the imaginary part σ″ (=ωε′) exhibits a local minimum give the PEM conductivity, σ. Examples of EIS data are shown in Figure S2. The estimated error of the conductivity measurement is approximately (±0.005 S/cm) based on our previous experience and propagating the uncertainty of the quantities in the measurement.

### Pulsed-Field Gradient Nuclear Magnetic Resonance

The diffusion coefficients of water in Nafion, PTPS, and SPES 40 (Figure 1) were measured by using a Bruker AVIII-850 spectrometer (Mannheim, Germany) equipped with a 5 mm microimaging probe. Membranes equilibrated at three different relative humidities (95, 75, 50%) were prepared using the exact same protocol in our previous paper, which is similar to the FTIR sample preparation.^45^ For the measurement, a stimulated echo (STE) pulse was placed on a gradient pulse. For the STE pulse, a relaxation delay of 10 s was applied. For the gradient pulse, a spoil gradient with a duration of 1 ms was chosen. For each measurement, 16 gradient pulse strengths were chosen from 0 to the maximum strength, and the maximum strength varied from 150 to 1400 G/cm. The diffusion coefficient was measured at various diffusion times from 10 to 600 ms for each sample. The diffusion coefficients were obtained by fitting the intensity decay of the water peak as a function of increasing pulse-field gradient strength with Stejskal–Tanner equation.^46^

### NMR *T*_1_ and *T*_2_ Relaxation

The *T*_1_ and *T*_2_ relaxation of D_2_O in Nafion, PTPS, and SPES 40 was measured on a Bruker AVIII-600 (Mannheim, Germany) spectrometer using ^2^H NMR. The samples at three different relative humidities were prepared using the same protocol as the PFG-NMR measurement. *T*_2_ of water was measured using a spin echo pulse sequence, and *T*_2_ was obtained by fitting the intensity decay of the D_2_O peak as a function of time using
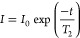
where *I*_0_ is the relative intensity at time 0, and *I* is the relative intensity at time *t*. *T*_1_ was measured using an inversion recovery pulse, and it was obtained by fitting the intensity increase of the D_2_O peak as a function of time using the following equation:
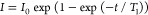
where *I*_0_ is the relative intensity at time 0, and *I* is the relative intensity at time *t*.

## Results and Discussion

The superacidic polymer PTPS was synthesized via polycondensation. The successful synthesis of the TFTS monomer and PTPS polymer was confirmed by ^1^H NMR and ^19^F NMR as shown in Figure S1. The IEC of the resulting polymer is 1.57 mequiv/g compared to an IEC of Nafion at 0.91 mequiv/g and an IEC for SPES 40 of 1.49 mequiv/g.

**Figure 2:**
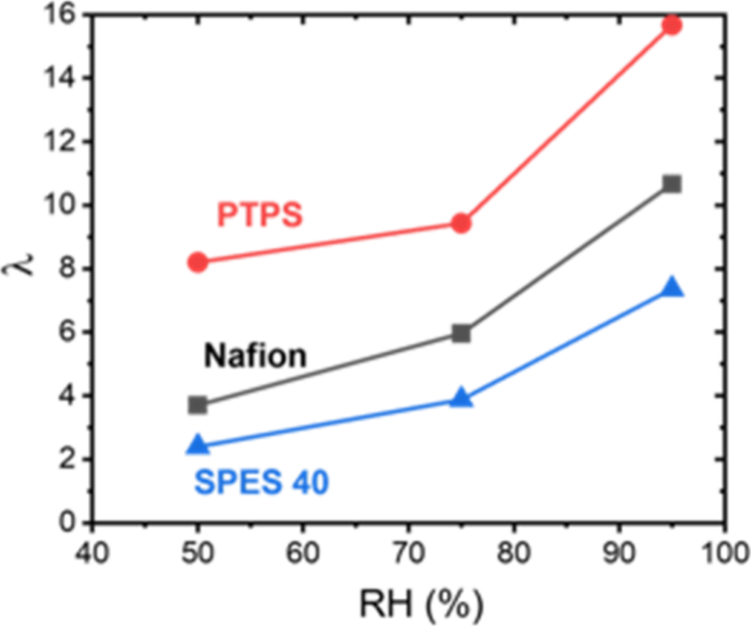
Hydration number as a function of relative humidity for Nafion, PTPS, and SPES 40.

The water uptake and IEC of Nafion, PTPS, and SPES 40 are summarized in Table S1, and the hydration number was calculated based on these values and is shown in Figure 2. At a fixed relative humidity, SPES 40 has a lower hydration number compared with the other two superacidic polymers likely due to the lower acidity of its acidic side chains and lower electron density of the sulfonate headgroup. This result is similar to previous work on superacidic polymers.^31^ Additionally, PTPS has a higher hydration number compared with Nafion due to the lower hydrophobicity of its partially fluorinated main chain compared to that of the perfluorinated Nafion main chain.

To study the water environment in each type of PEM material, we analyzed the OD stretch peak in the FTIR spectra under various RH conditions. In this study, we used 15% HOD in H_2_O, which is a higher concentration than used previously (typically, <6%) to enhance the OD stretch peak intensity.^37^ However, it was confirmed that the peak position and full width at half-maximum (fwhm) of 15% HOD (2511 and 156 cm^–1^; see Figure S3) are similar to those of 6% HOD (2507 and 151 cm^–1^).

The typical trend of OD stretch peak positions of water in sulfonic acid-containing polymer membranes is that the stronger water interactions with sulfonic acid result in higher wavenumber OD stretch peak positions.^37,39−41^ This effect is due to the hydrogen bond between water and the sulfonic acid anion being longer and, thus, weaker than that between bulk water molecules, leading to a higher O–D bond vibrational frequency.

**Figure 3:**
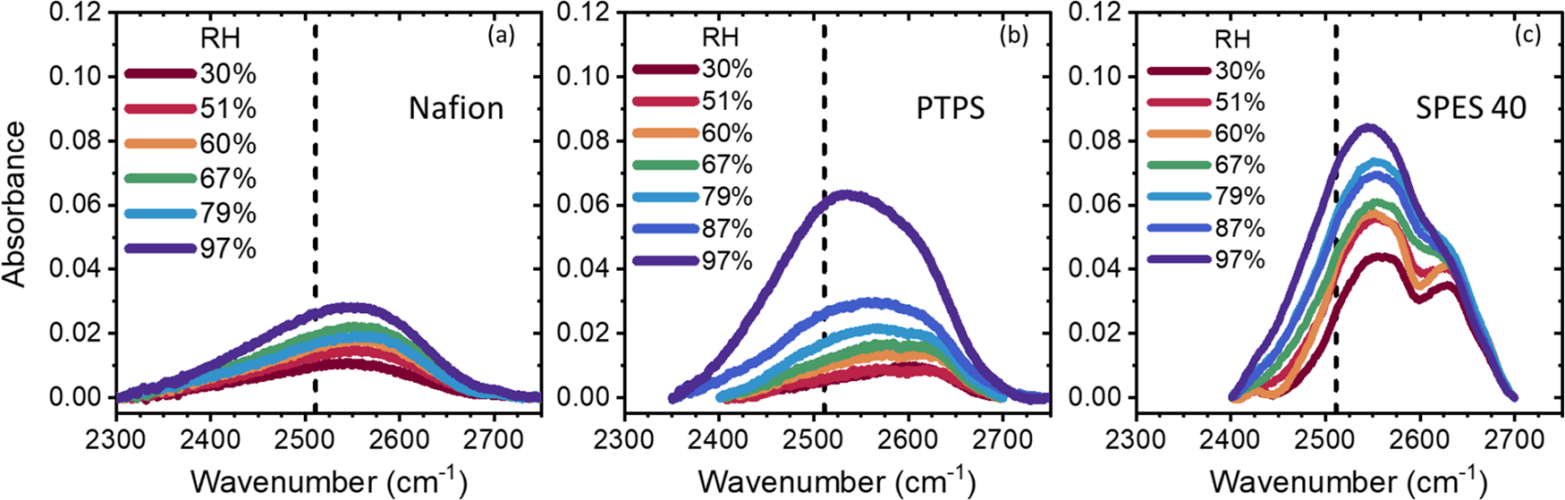
OD stretch peak of: (a) Nafion (NR211), (b) PTPS, and (c) SPES 40 under various RH conditions at room temperature. The vertical dashed lines indicate the OD stretch peak position of bulk water (∼2511 cm^–1^).

The OD stretch peaks of Nafion, PTPS, and SPES 40 at increasing RH are shown in Figure 3. For all materials, the overall peak area increased with increasing RH, resulting from the larger water uptake of the samples. Additionally, the peak shape and position of the OD stretch were strongly dependent on the type of PEM, indicating different water environments across the samples. All materials showed a blue shift of the overall peak position compared to bulk water (∼2511 cm^–1^) resulting from the perturbed H-bond network due to the association of the water molecules and sulfonic acid moieties.

**Figure 4:**
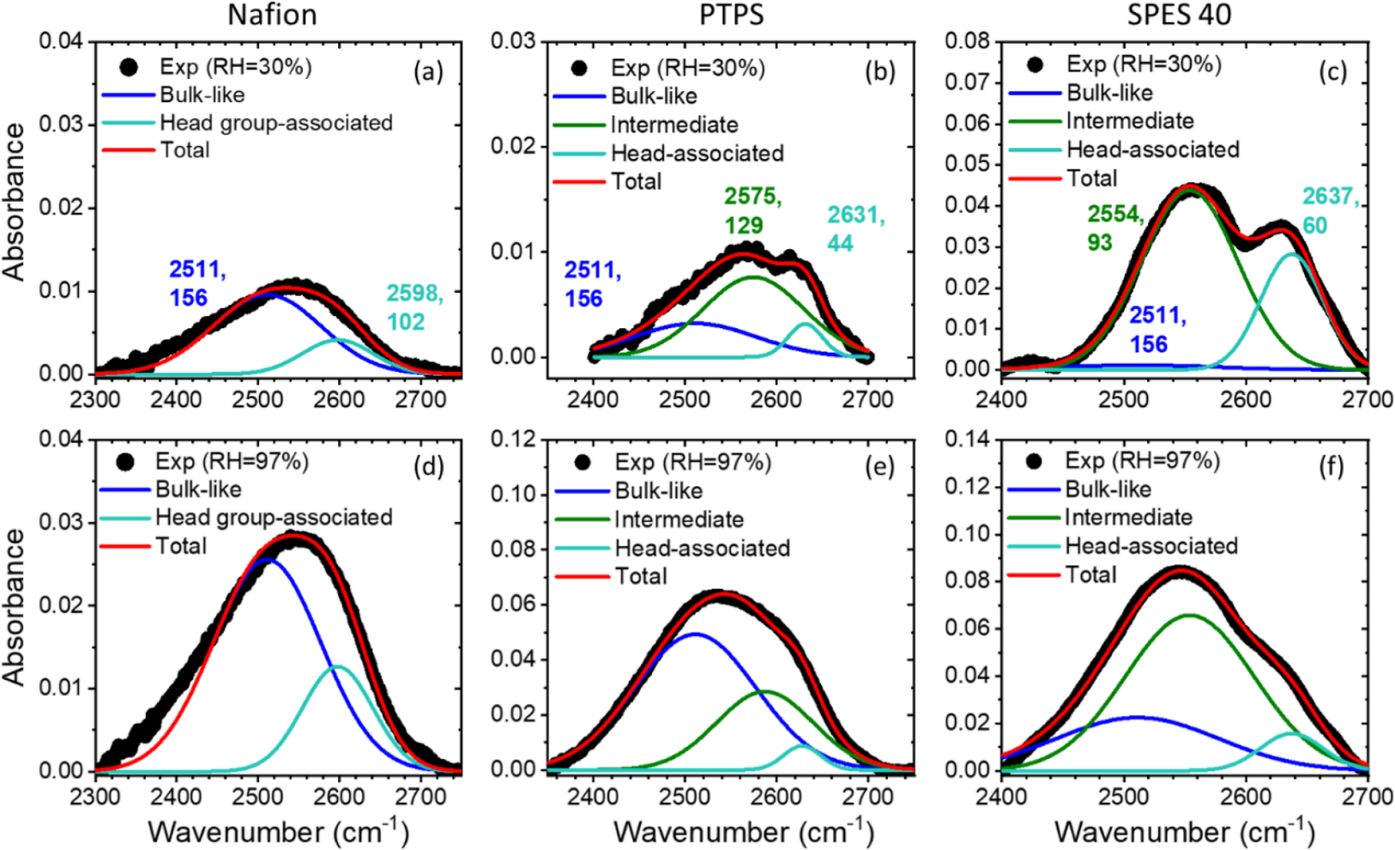
Representative examples of deconvolution of OD stretch peak using 2-component for: (a, d) Nafion and 3-component for (b, e) PTPS and (c, f) SPES 40. The black square symbol is the experimental FTIR data at (a–c) RH = 30% and (d–f) RH = 97%. The numbers in (a)–(c) indicate the peak center and full width at half-maximum (fwhm) of each water component.

OD stretch peaks were deconvoluted using multiple Gaussian peaks to analyze the water environments in more detail.^41,47−49^Figure 4 shows the representative fitting results depending on the RH condition and material type. At low RH, e.g., 30%, water molecules are associated with sulfonic acid groups or bulk water, giving a peak that is representative of headgroup-associated and intermediate water. Therefore, we can characterize peak parameters of those interfacial water populations with the data at 30% RH, including a bulk water peak, as needed. The detailed results are shown in Figure 4a–c. The peak center and fwhm of bulk-like water were fixed to the measured values of bulk 15% HOD in H_2_O, and the peak parameters (center and fwhm) for the other two peaks were set to vary freely. The intensity of each peak, which is related to the amount of each water phase, was also allowed to vary to obtain the best fit.

In the case of Nafion, Moilanen et al.^37^ reported that there are two populations of water: (1) headgroup-associated and (2) bulk-like water. Consistently, we also successfully fitted the OD spectra of Nafion with two Gaussian peaks: one peak for headgroup-associated water and the second peak for bulk-like water. In Nafion, no intermediate water was observed.

Three Gaussian peaks were required when fitting the spectra of aromatic backbone-based materials with tethered arylsulfonates, as reported previously.^39−41^ The three water environments are bulk-like, intermediate, and headgroup-associated water.

The peak center of headgroup-associated water is blue-shifted by 33–39 cm^–1^ for aromatic-based materials (PTPS and SPES 40) compared to Nafion, similar to what was observed previously,^39−41^ indicating that the interaction strength between the sulfonate group and water molecules is weaker for Nafion. The stronger acidity of the sulfonic acid group in Nafion, due to the strong electron-withdrawing effects of the perfluorinated tether leads to low charge density at the headgroup, forming weaker H-bonds with water.

Using the fitted values of the peak center and fwhm of the OD stretch of each water environment at RH = 30%, the data for higher RH conditions can also be deconvoluted, while the relative intensity of each peak was freely varied. For example, the peak center and fwhm values of both bulk-like and headgroup-associated water were held constant in the case of Nafion. Meanwhile, in the case of PTPS and SPES 40, the fwhm of the intermediate water peak was freely varied as we fitted the data at higher RH conditions, while all other peak parameters (peak center and fwhm) of bulk-like and headgroup-associated water were fixed. The peak center of intermediate water was also fixed at the values characterized at RH = 30% in most cases, with the exception of RH = 87 and 97% for PTPS. The representative fitting results at RH = 97% with good fitting quality are shown in Figure 4d–f. All the other fitting results and parameters at different RH conditions can be found in Figures S4–S6 and Tables S2–S4 in Supporting Information.

We applied the non-Condon correction for peak area prior to evaluating the relative amounts of each type of water.^47,50^ This correction is required because the transition dipole strength (μ) increases with decreasing frequency and the absorbance in linear IR spectroscopy is affected by μ^2^.^51^ Thus, the frequency-dependent μ was corrected using
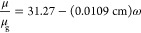
1
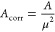
2where μ_g_ and ω are the gas-phase transition dipole and peak frequency, respectively. Then, the corrected peak area, *A*_corr_, can be calculated from the fitted peak area, *A*, using μ^2^ as expressed in eq 2.

**Figure 5:**
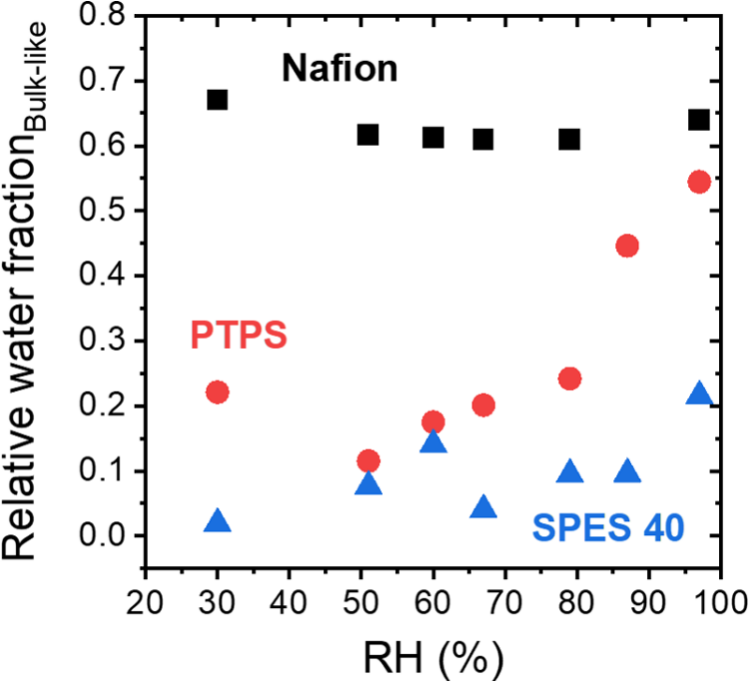
Relative bulk-like water fraction of Nafion (black square), PTPS (red circle), and SPES 40 (blue triangle) as a function of RH.

Finally, the relative fraction of each water phase was obtained using the corrected peak areas in Figure 5. Bulk-like water fraction increased with increasing RH for PTPS and SPES 40, while Nafion shows a relatively constant value at ∼0.61–0.67. Generally, the relative fraction of bulk-like water is greatest in Nafion, followed by PTPS, and then SPES 40. This trend is related to the material-dependent interaction between sulfonic acid group and water molecules. Nafion and PTPS have strong electron-withdrawing fluorine atoms adjacent to the sulfonate, resulting in low charge density of the acidic headgroup. This difference in acidity leads to weaker interaction between sulfonic acid groups and water molecules, forming a larger fraction of bulk-like water in the systems. The introduction of aromatic rings into the polymer backbone weakens phase segregation strength and stiffens the backbone, restricting the formation of concentrated ionic domains such that Nafion has a higher bulk-like water fraction compared to PTPS. Finally, because SPES 40 has less organized ionic domains due to the backbone stiffness and does not have a strong electron-withdrawing group such as fluorine to increase the acidity of the tethered acid, this polymer has a lower fraction of bulk-like water compared to Nafion and PTPS, particularly at higher relative humidity values.

**Figure 6:**
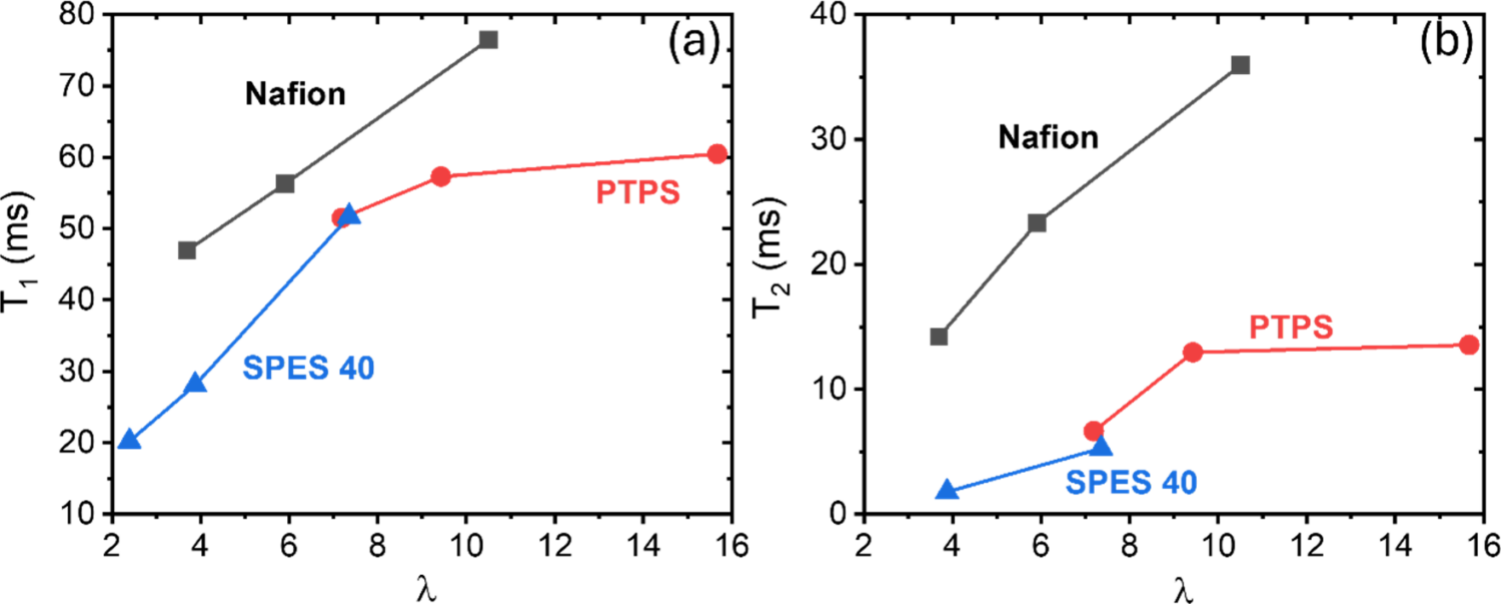
(a) *T*_1_ and (b) *T*_2_ of D_2_O for Nafion, PTPS, and SPES 40.

*T*_1_ and *T*_2_ data for water molecules in the three types of PEMs from this study are shown in Figure 6. *T*_1_ and *T*_2_ represent the degree of interaction between water molecules and the membrane, where *T*_1_ is the longitudinal relaxation (*z*-axis) and *T*_2_ is the transverse relaxation (*xy*-plane).^52^ As demonstrated by previous experimental and theoretical work, stronger interactions between water and PEMs lead to a faster nucleus relaxation time, thus decreasing the observed *T*_1_ and *T*_2_ values.^31,53,54^ Nafion shows a longer relaxation time than the aromatic backbone polymers due to weaker water-sulfonic acid interactions and its distinct phase-separated structure. As discussed previously, due to the strong electron-withdrawing characteristics of the perfluorinated moieties, the sulfonate groups on Nafion and PTPS have lower electron density than in the aromatic sulfonic acid of SPES, thus leading to a weaker interaction between the ionic headgroup and water molecules.^55^ PTPS has a lower relaxation time compared with that of Nafion. This observation is likely due to a relatively lower degree of phase segregation in the PTPS material due to the rigidity of its aromatic-based polymer main chain. This result agrees well with the water environment analysis (Figure 5) using FTIR that the interaction between water and PTPS is between that of Nafion and SPES 40.

**Figure 7:**
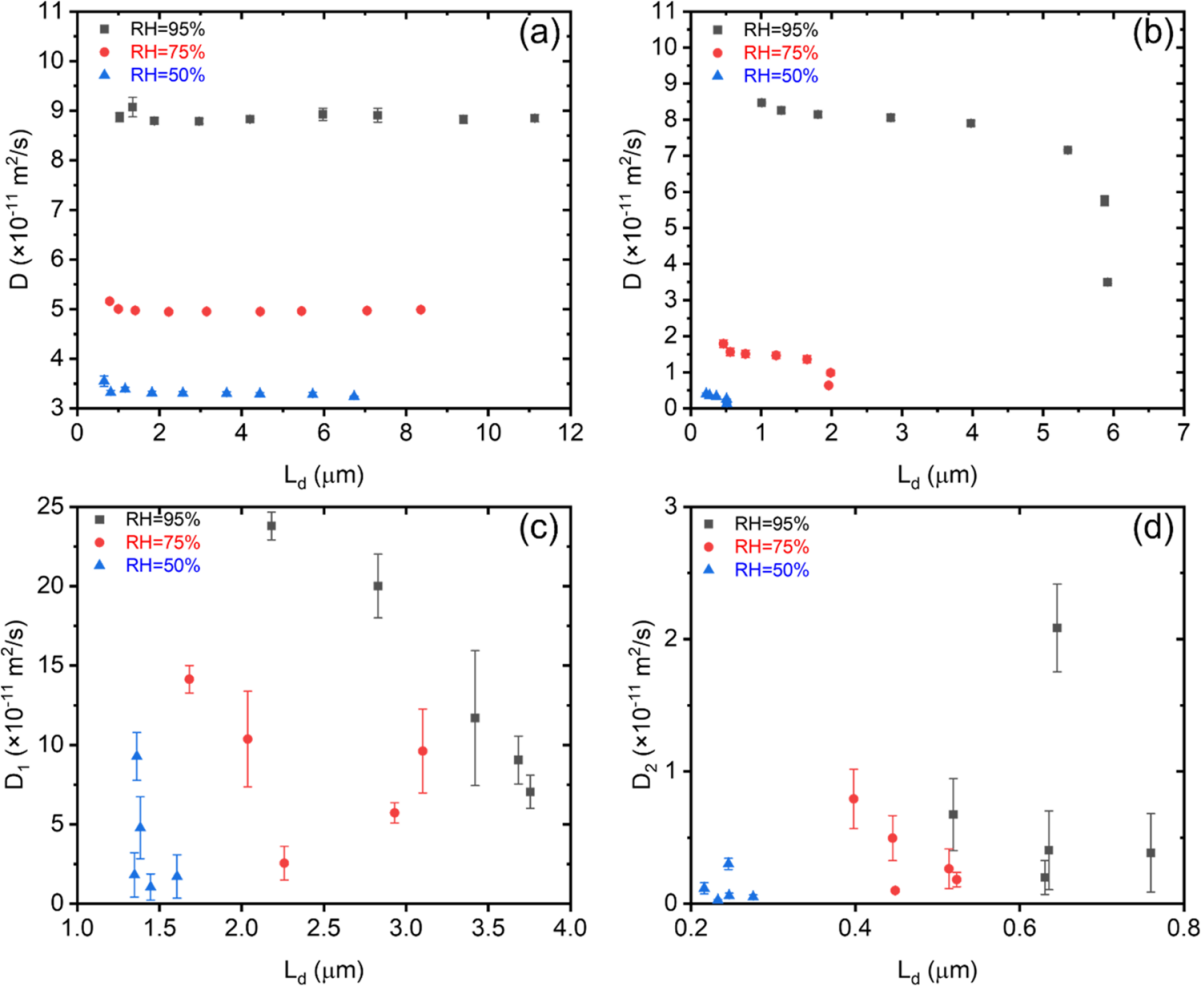
Diffusion coefficient as a function of diffusion length  for (a) Nafion; (b) SPES 40; (c) *D*_1_ component of PTPS; and (d) *D*_2_ component of PTPS.

To better understand the water dynamics over larger length scales, the water diffusion coefficient of hydrated Nafion, SPES 40, and PTPS was measured at various diffusion times (Δ), which controls the length scale of the diffusion coefficient measurement.^56^ The diffusion coefficient of Nafion is independent of the diffusion times measured as is shown in Figure 7, due to the formation of interconnected water channels.^57^ However, both SPES 40 and PTPS show a systematic decrease in the diffusion coefficient with increasing diffusion time. A similar trend has been observed by Thieu et al.^32^ where they hypothesized that this decrease is due to both the increase in the interaction between water and the polymer and the heterogeneous morphological restrictions at larger length scales.

Additionally, as shown in Figures S7 and 7, PTPS has two diffusion processes. This phenomenon needs further investigation as it has not been widely reported for these types of materials. Our assumption is that these two diffusion coefficients correspond to the water diffusion motion for different water species identified by FTIR. The smaller diffusion coefficient corresponds to the diffusion of the headgroup-associated water, while the larger diffusion coefficient corresponds to the diffusion of intermediate and bulk water species.

**Figure 8:**
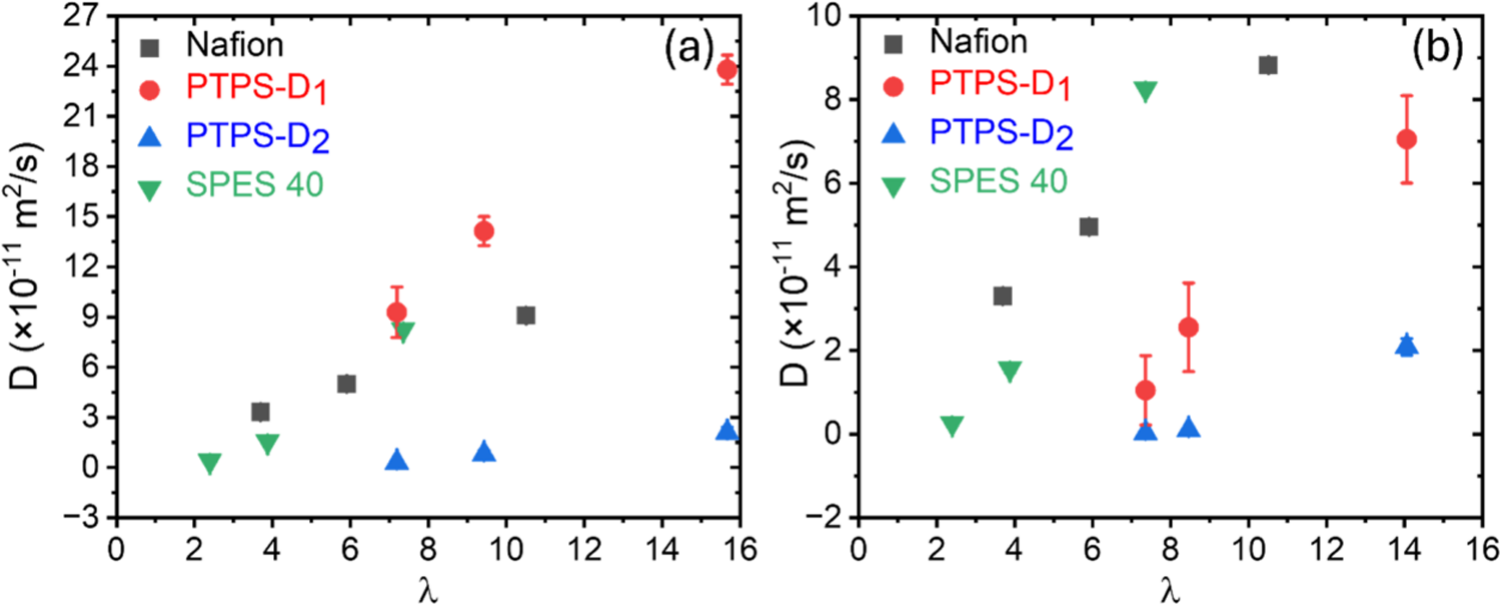
Diffusion coefficient of Nafion, PTPS, and SPES 40 at: (a) Δ = 10 ms, and (b) Δ = 100 ms.

The diffusion coefficients of water in Nafion, PTPS, and SPES 40 at Δ = 10 and 100 ms are shown in Figure 8. At short diffusion lengths of approximately 1 μm (Δ = 10 ms, Figure 8a), the trend of the diffusion coefficient roughly agrees with the *T*_1_ and *T*_2_ results (Figure 6). However, at longer diffusion lengths of ∼3 μm (Δ = 100 ms, Figure 8b), due to the impact of restricted diffusion due to expected morphological differences, PTPS showed the lowest diffusion coefficient compared with Nafion and SPES 40.

**Figure 9:**
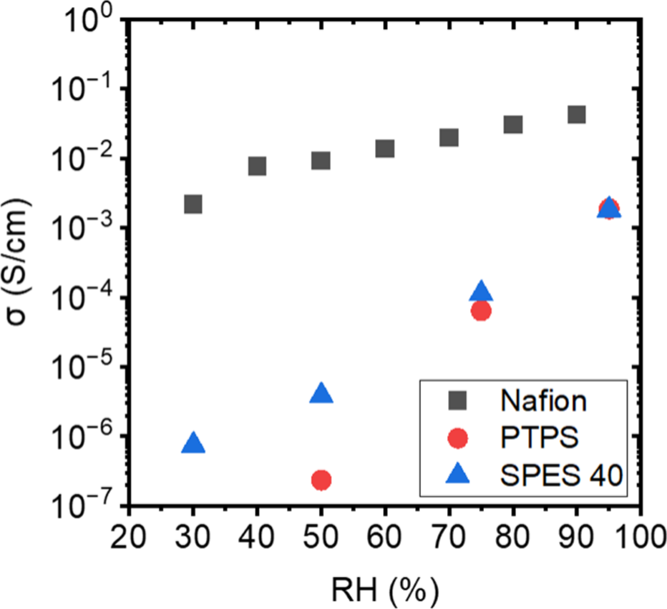
Proton conductivity of Nafion (black square), PTPS (red circle), and SPES 40 (blue triangle) at *T* = 25 °C as a function of RH.

To further understand the relationship between the microscopic water dynamics and macroscopic proton conductivity, the proton conductivities of Nafion, SPES 40, and PTPS were measured, Figure 9. Nafion showed higher proton conductivity than SPES 40 at all RH conditions. This suggests that Nafion’s higher fractions of bulk-like water and weaker H-bond strength with sulfonic acid groups facilitate proton transport compared to SPES 40. It is also consistent with *T*_1_, *T*_2_, and diffusion coefficient results probed by NMR experiments. While the proton conductivity of PTPS and SPES 40 are similar at 95% RH, the conductivity of PTPS decreases more strongly with % RH than SPES 40. This trend agrees well with the diffusion coefficient at Δ = 100 ms (Figure 8b). Therefore, the conductivities of both PTPS and SPES 40 are strongly restricted by proton diffusion at larger length scales. We attribute these properties to poorly connected hydrophilic domains, which are the result of limited backbone flexibility.

## Conclusions

In this research, a polymer with an aromatic main chain and a superacid side chain (PTPS) was synthesized and characterized. Together with Nafion and SPES 40, the water dynamics of PTPS were measured at varying length scales using FTIR and NMR measurements. FTIR and *T*_1_ and *T*_2_ relaxation times from NMR agreed qualitatively and demonstrated that the water in PTPS is associated with the ionic site at a level that is intermediated between SPES 40 and Nafion. At larger length scales, restricted dynamics are apparent in PTPS, specifically water diffusion coefficient measured by PFG-NMR and proton conductivity measured by EIS were lower relative to Nafion. This type of work where the chemical structure, conductivity, and water dynamics are connected across different materials classes can pave the way for further developing PEMs for a range of electrochemical applications. This study demonstrates the importance of both the acid strength and backbone flexibility in producing high proton conductivity in hydrated polymers.

## Supporting Information Available

NMR spectroscopy of PTPS, water uptake, and IEC of Nafion, SPES 40, and PTPS, FTIR OD stretch peak deconvolution results (PDF)
