# Supplementary material for: Water Dynamics of Superacid Aromatic Proton Exchange Membranes for Fuel Cell Applications
Source: Macromolecules. 2025 Feb 20;58(5):2630–9. doi: 10.1021/acs.macromol.4c02925 (PMC11912528; doi:10.1021/acs.macromol.4c02925)
Supplement: Supplementary file 1 — ma4c02925_si_001.pdf [file ma4c02925_si_001.pdf]

Supporting Information for:

Water Dynamics of Superacid Aromatic Proton Exchange Membranes for Fuel Cell Applications

Zitan Huang[‡1], *Sol Mi Oh*[‡2], *Karen I. Winey*[*,2,3], *Michael A. Hickner*[*,4]

[1]Department of Materials Science and Engineering, The Pennsylvania State University, University Park, Pennsylvania 16802, United States.

[2]Department of Materials Science and Engineering, University of Pennsylvania, Philadelphia, Pennsylvania 19104, United States

[3]Department of Chemical and Biomolecular Engineering, University of Pennsylvania, Philadelphia, Pennsylvania 19104, United States

[4]Department of Chemical Engineering and Materials Science, Michigan State University, East Lansing, Michigan 48824, United States

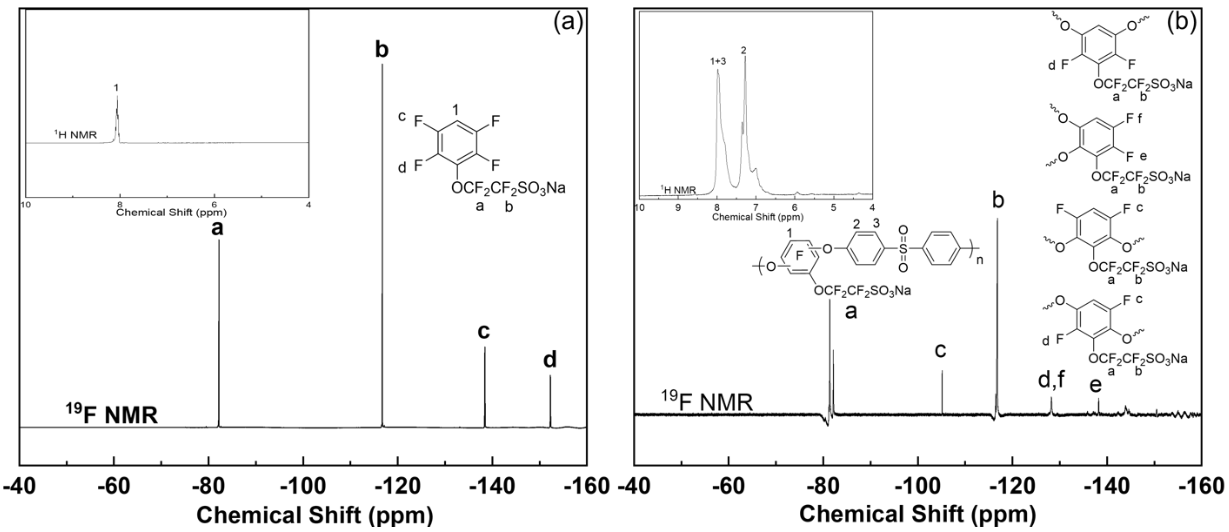

Figure S1. ^{19}F NMR and ^{1}H NMR of: (a) sodium 1,1,2,2-tetrafluoro-2-(2,3,5,6-tetrafluorophenoxy) ethane-1-sulfonate, and (b) PTPS.

The successful synthesis of both monomer and PTPS polymer was confirmed by ^{19}F NMR and ^{1}H NMR as is shown in Figure S1. Due to the similarity in reactivity of the four electron-deficient carbons on sodium 1,1,2,2-tetrafluoro-2-(2,3,5,6-tetrafluorophenoxy) ethane-1-sulfonate, the aromatic substitution reaction can occur on any of these positions, leading to mixed linkages in the backbone structure.

Table S1. Water uptake and IEC of Nafion, SPES 40 and PTPS.

	Nafion RH (%)			SPES 40 RH (%)			PTPS RH (%)		
	50	75	95	50	75	95	50	75	95
Water Uptake (wt %)	6	10	18	6	10	20	23	27	44
IEC (meq/g)	0.91			1.49			1.57 (NMR)/1.51 (Titration)		

The water uptake and IEC of Nafion, SPES 40, and PTPS are shown in **Table S1**. The hydration number in **Figure 2** (λ), is calculated from the value above using the following equation:

$$\lambda = Water\ uptake \times \frac{1000}{IEC \times 18}$$

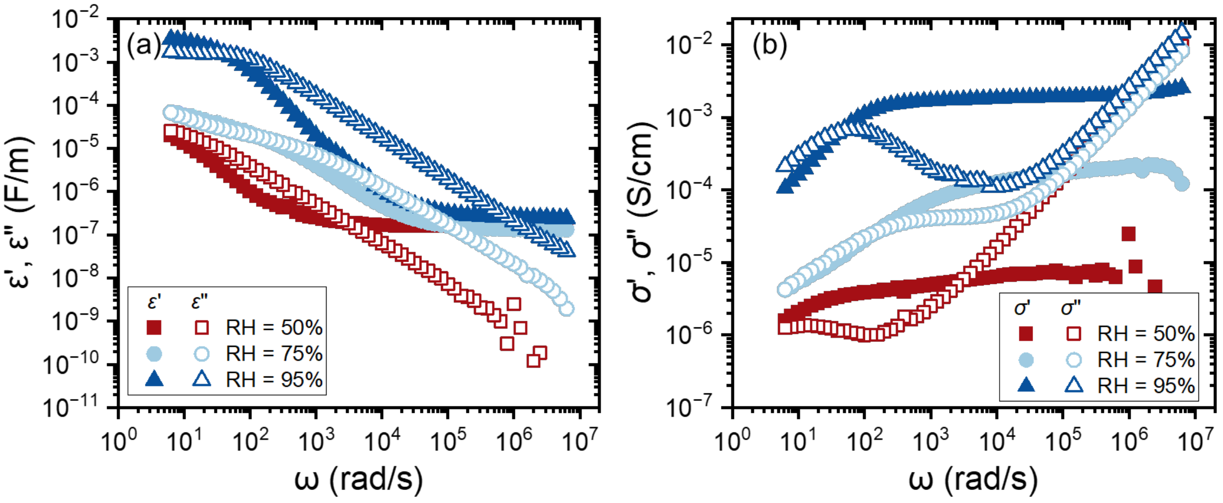

Figure S2. Examples of electrochemical impedance spectroscopy (EIS) results for SPES 40: (a) the measured real (ε') and imaginary (ε'') parts of the complex dielectric constant, and (b) the converted real (σ') and imaginary (σ'') parts of complex conductivity as a function of angular frequency, ω.

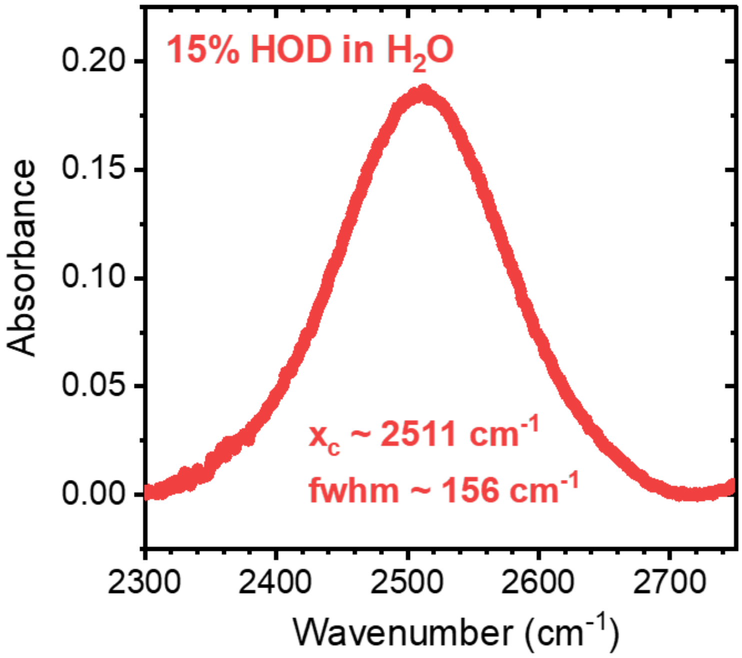

Figure S3. OD stretch peak of bulk 15% HOD in H2O. x_c and fwhm indicate the peak center and full-width-half-maximum of a Gaussian-fitted peak.

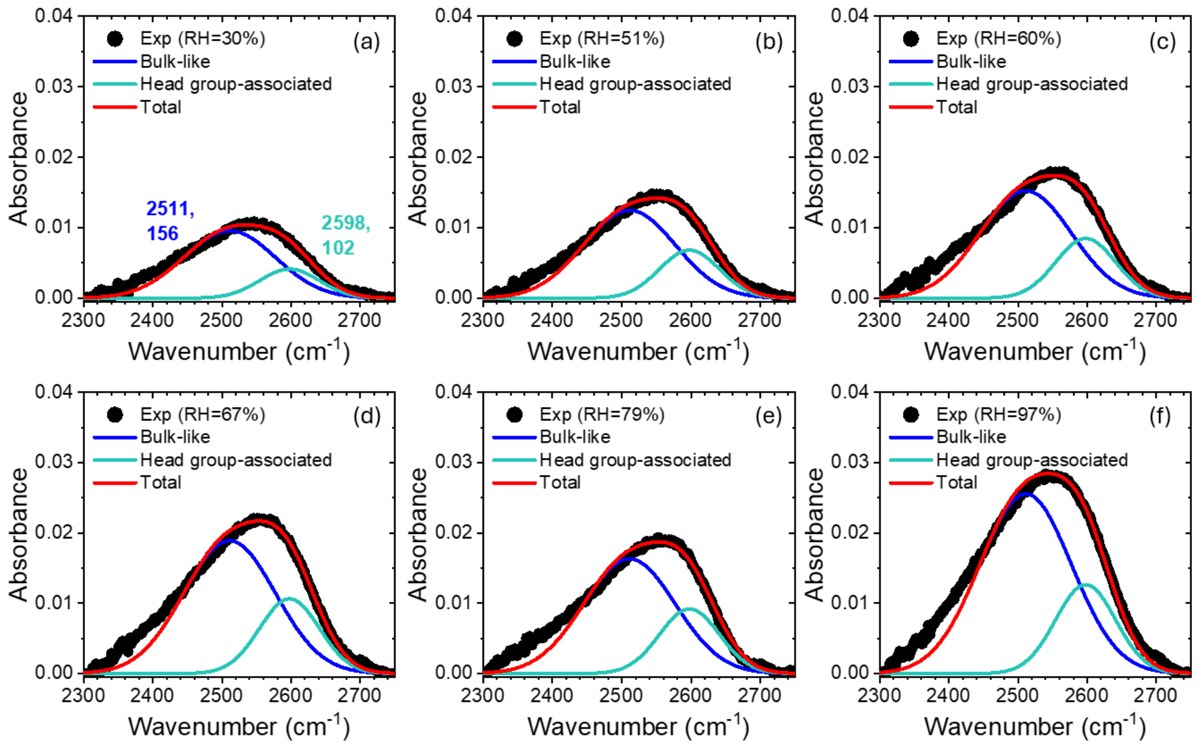

Figure S4. OD stretch peaks for Nafion under various RH conditions. Black circles represent the experimental OD stretch peak and blue, green-blue, and red curves indicate the deconvoluted bulk-like water, headgroup-associated water, and total peak fit results. The first and second numbers in (a) are the center and fwhm of each water environment.

Table S2. Fitted peak parameters of water populations for Nafion.

**RH**		**30%**	**51%**	**60%**	**67%**	**79%**	**97%**
Headgroup-associated	Peak center (cm^{-1})	2598	2598	2598	2598	2598	2598
	fwhm (cm^{-1})	102	102	102	102	102	102
Bulk-like water	Peak center (cm^{-1})	2511	2511	2511	2511	2511	2511
	fwhm (cm^{-1})	156	156	156	156	156	156

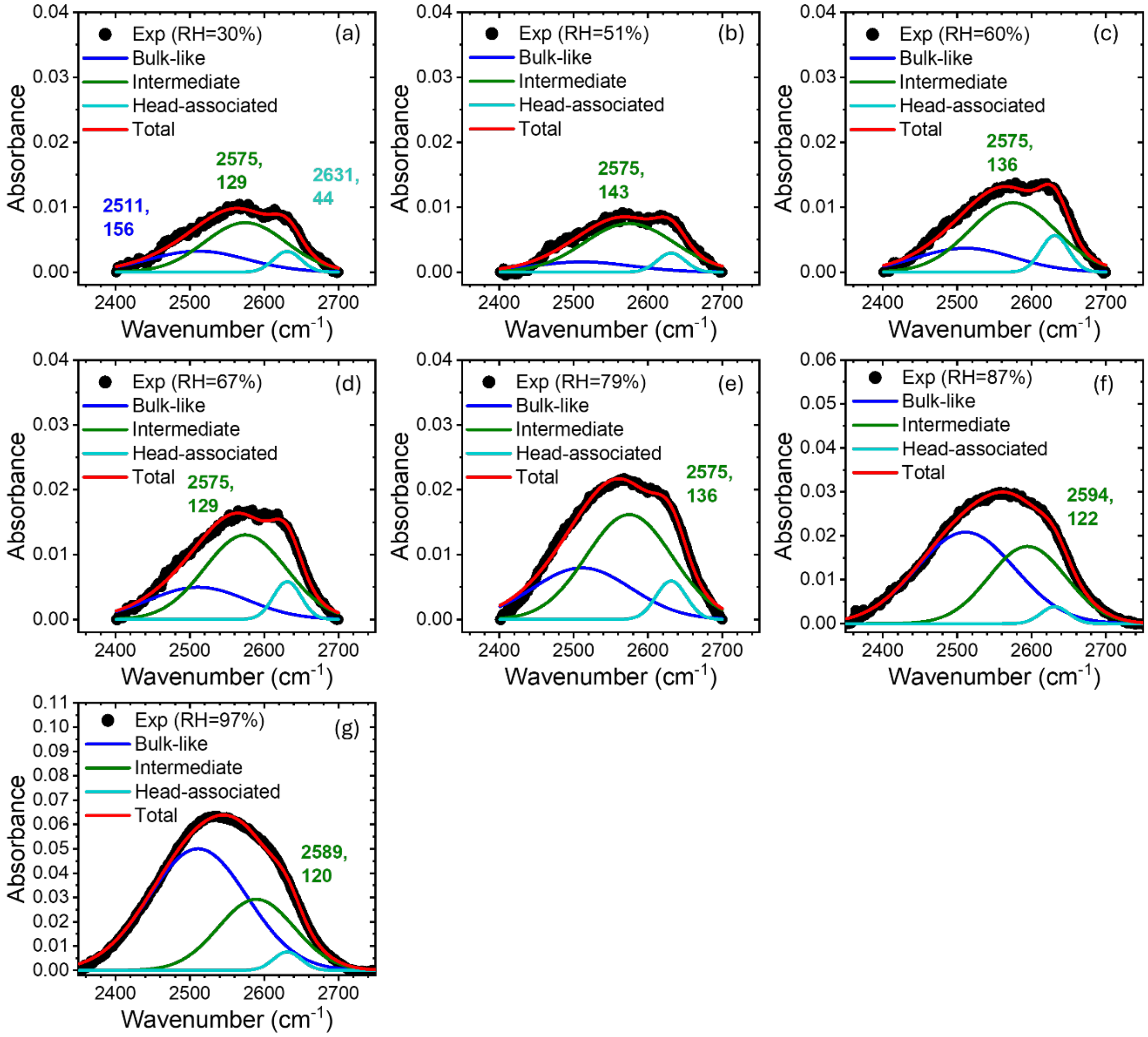

Figure S5. OD stretch peaks for PTPS under various RH conditions. Black circles represent the experimental OD stretch peak and blue, green, green-blue, and red curves indicate the deconvoluted bulk-like water, intermediate water, headgroup-associated water, and the total peak fit results. The first and second numbers in (a) are the center and fwhm of each water environment. At RH higher than 30% ((b)-(g)), the numbers in the graph are the peak center and fwhm values of the intermediate water.

Table S3. Fitted peak parameters of water populations for PTPS.

RH		30%	51%	60%	67%	79%	87%	97%
Headgroup-associated	Peak center (cm^{-1})	2631	2631	2631	2631	2631	2631	2631
	fwhm (cm^{-1})	44	44	44	44	44	44	44
Intermediate water	Peak center (cm^{-1})	2575	2575	2575	2575	2575	2594	2589
	fwhm (cm^{-1})	129	143	135	129	136	122	120
Bulk-like water	Peak center (cm^{-1})	2511	2511	2511	2511	2511	2511	2511
	fwhm (cm^{-1})	156	156	156	156	156	156	156

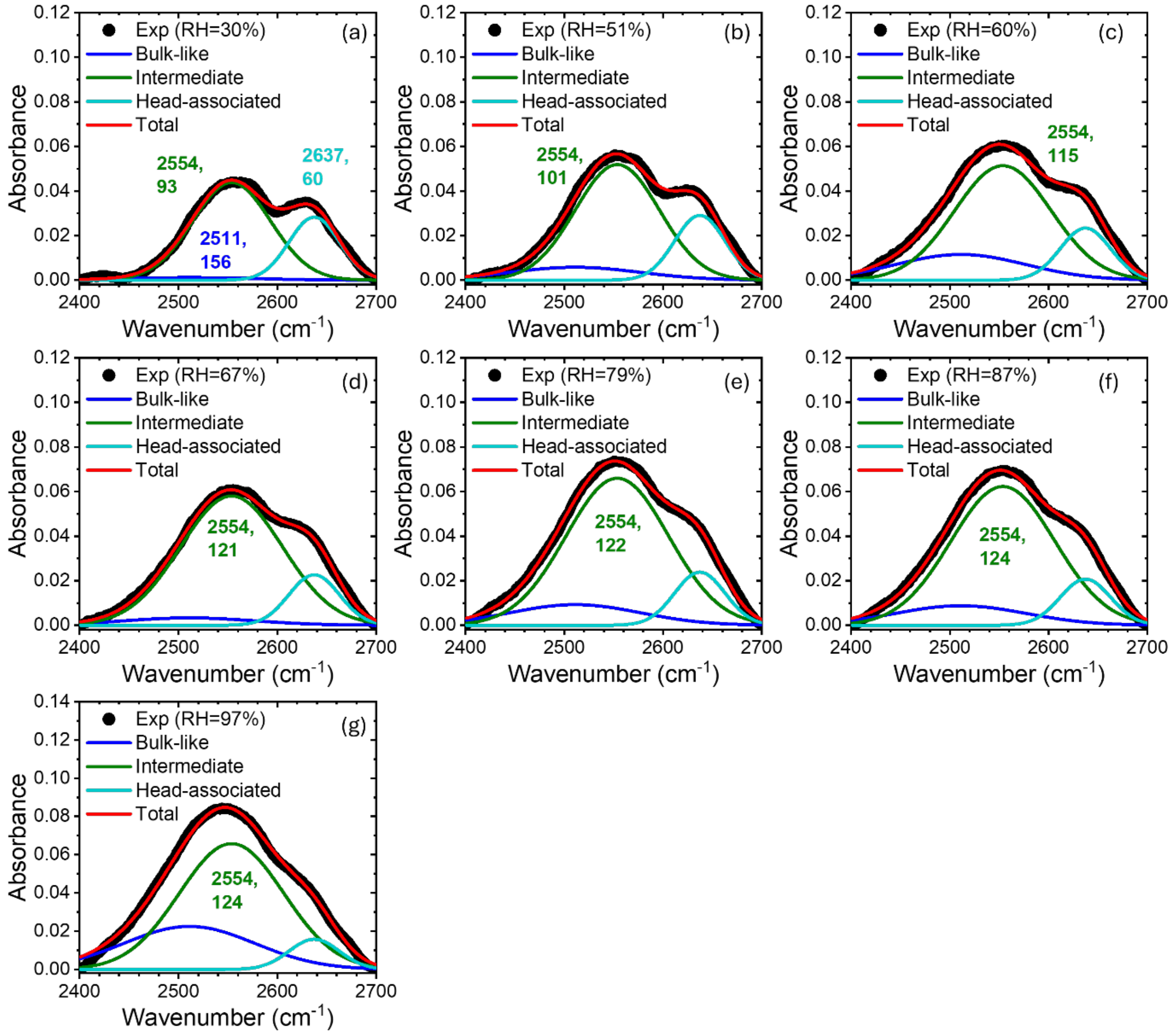

Figure S6. OD stretch peaks of SPES 40 under various RH conditions. Black circles represent the experimental OD stretch peak and blue, green, green-blue, and red curves indicate the deconvoluted bulk-like water, intermediate water, headgroup-associated water, and total fitting results. The first and second numbers in (a) are the center and fwhm of each water phase. At RH conditions higher than 30% ((b)-(g)), the numbers in the graph are about the peak center and fwhm values of the intermediate water.

Table S4. Fitted peak parameters of water populations for SPES 40.

RH		30%	51%	60%	67%	79%	87%	97%
Headgroup-associated	Peak center (cm^{-1})	2637	2637	2637	2637	2637	2637	2637
	fwhm (cm^{-1})	60	60	60	60	60	60	60
Intermediate water	Peak center (cm^{-1})	2554	2554	2554	2554	2554	2554	2554
	fwhm (cm^{-1})	93	101	115	121	122	124	124
Bulk-like water	Peak center (cm^{-1})	2511	2511	2511	2511	2511	2511	2511
	fwhm (cm^{-1})	156	156	156	156	156	156	156

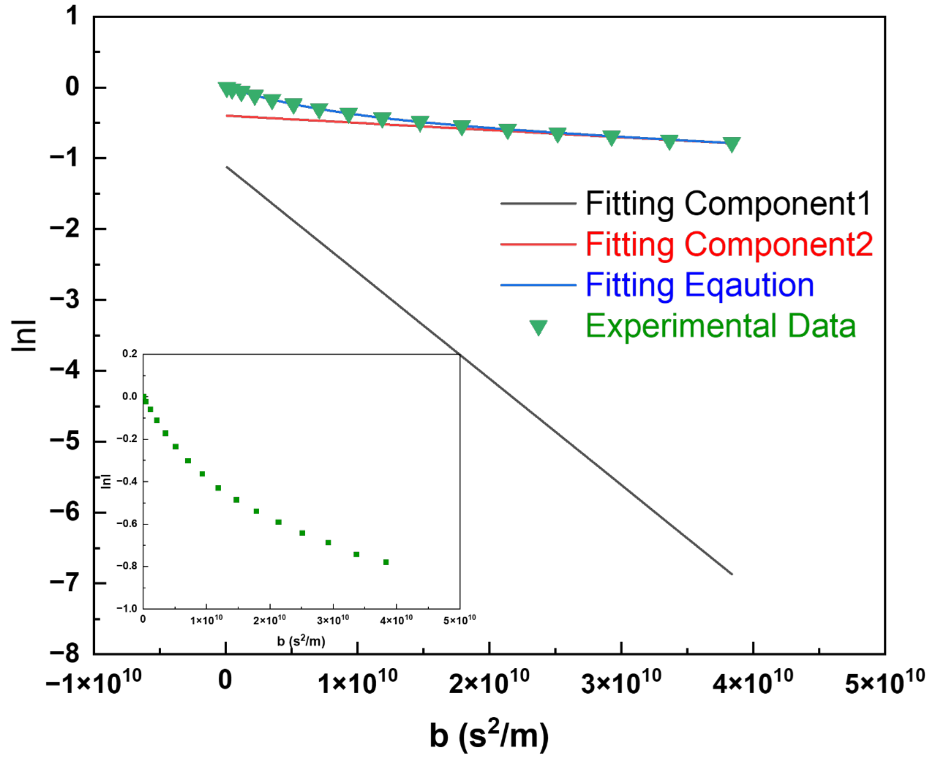

Figure S7. PFG-NMR Result fitting for water in PTPS at RH=75%, Δ=10 ms.

As is shown in the inset of **Figure S7**, it is not possible to fit the intensity data using a one component Stejskal−Tanner equation. Therefore, the obtained data was fitted with the following two component Stejskal−Tanner equation:

$$lnI = \ln\,(I_{0,1}\exp(-D_1 b) + I_{0,2}\exp(-D_2 b))$$

where I is the spin-echo intensity at a certain gradient strength, $I_{0,1}$ and $I_{0,2}$ are the intensity at zero gradient for fitting component 1 and fitting component 2, b is the Stejskal−Tanner factor that is proportional to the gradient strength applied, D_1 and D_2 are fitted diffusion coefficient for component 1 and component 2.
